# The Correlation of Fetal Kidney Length With Gestational Age From 24 Weeks of Pregnancy

**DOI:** 10.7759/cureus.77687

**Published:** 2025-01-19

**Authors:** Vinod Kumar, Jayalakshmy MD, Bimal John, Niveditha Kartha, Nimmi Varghese, Sreekumari Radha

**Affiliations:** 1 Obstetrics and Gynaecology, Credence Hospital, Thiruvananthapuram, IND; 2 Biostatistics, Credence Hospital, Thiruvananthapuram, IND; 3 Obstetrics and Gynaecology, Government Medical College, Thiruvananthapuram, Thiruvananthapuram, IND; 4 Obstetrics and Gynaecology, Stoke Mandeville Hospital, Aylesbury, GBR

**Keywords:** biometric indices, correlation, fetal kidney length, gestational age, investigation, prediction, regression, ultrasound

## Abstract

Background

There has been an ongoing quest for a single ultrasonographic parameter to assess the accurate gestational age (GA) of the fetus that is unaffected by intrauterine growth restriction (IUGR). While fetal kidney length (FKL) is a parameter that can be used for dating late pregnancies, it has not been studied extensively as a biometric index for GA estimation. This study aimed to assess FKL in pregnancy from 24 weeks and its correlation with the GA of the fetus.

Materials and methods

We conducted a cross-sectional study in the Department of Obstetrics and Gynecology at Credence Hospital, Thiruvananthapuram, Kerala, India over six months. Ultrasounds were performed and mean FKL (average length of right and left kidney), biparietal diameter (BPD), head circumference (HC), abdominal circumference (AC), and femur length (FL) were measured by a single experienced sonologist.

Results

Among the five fetal biometric indices, we found that FKL displayed the strongest correlation with GA (r=0.981). FKL was also the most accurate parameter in predicting GA, with a standard error of 9.83 days. The most inaccurate one was head circumference, with a standard error of 14.05 days. The stepwise regression analysis showed that the most precise means of estimating GA was a model that included all five variables, with an accuracy of 8.09 days.

Conclusions

Our findings showed that by measuring FKL, pregnancies can be dated within 9.83 days among people who booked late or those who had forgotten the date of their last menstrual period.

## Introduction

Assessment of the gestational period is the cornerstone of providing quality maternity care. It is critical to prevent iatrogenic pre-maturity or post-maturity, which can result in perinatal complications. The correct estimation of the period of gestation is more important in high-risk pregnancies, such as those that involve gestational hypertensive disorders, growth restriction, and gestational diabetes mellitus, or when the termination of pregnancy is planned.

The obstetric ultrasound scan is of crucial importance in accurately determining the period of gestation. The composite gestational age (GA) in the second and third trimesters is assessed most commonly using the Hadlock method [1], which uses the biparietal diameter (BPD), head circumference (HC), abdominal circumference (AC), and femur length (FL) of the fetus. Other parameters used include the transcerebellar diameter [2], clavicular length [3], and foot length [4].

However, most of these methods become less reliable with variability of size in relation to age [5]. Some recent studies [6,7] have reported that the fetal kidney length (FKL) correlates well with the period of gestation. As with the development of organs during fetal life, there is progressive growth of the fetal kidneys as well. Though fetal kidneys show variation in terms of the width of anteroposterior dimensions, in conditions like fetal growth restriction (FGR), no significant variation has been found in FKL with any maternal medical conditions including obesity [8,9]. In addition, Several studies have shown that there is no significant difference between the length of the right and left kidneys in fetuses [10]. Also, fetal sex does not affect fetal kidney size [7].

The fetal kidney grows in length by 1.7 mm every 15 days during the entire pregnancy, even when there are underlying medical conditions like FGR [11]. Thus, it is a parameter that can be easily measured to date pregnancies [10]; there are scarce studies on this in our population. Hence, we believe that this study is unique in that it aims to measure FKL from 24 weeks of gestation and its correlation with the gestational age of the fetus. The accuracy of FKL was compared with other commonly used indices like BPD, HC, AC, and FL in dating pregnancy.

## Materials and methods

Study setting and ethical clearance

A cross-sectional study was conducted in the Department of Obstetrics and Gynecology (OB/GYN) of a multi-specialty hospital. The study adhered to the principles of the Helsinki Declaration. The study spanned six months, from August 1, 2022, to January 31, 2023. Ethical clearance for the study was obtained from the ethical committee of the institution (approval no: CH/CHIEC/03/2022, dated July 29, 2022). Participants gave informed consent before participation.

Sample size

Based on the results of a correlation coefficient analysis between FKL and gestational age (r=0.947) in a previous study [12] and with 2% precision and 95% confidence, a minimum of 108 samples were required. Subsequently, a total of 150 participants were recruited. using a simple random sampling method.

Inclusion criteria

The criteria for inclusion in the study were as follows: (1) low-risk antenatal women with no medical complications; (2) above 24 weeks of gestation; (3) regular periods with a known date of last menstrual period (LMP); (4) first-trimester ultrasound GA detected by crown-rump length; and (5) difference between GA detected by LMP and first-trimester ultrasound <5 days.

Exclusion criteria

The criteria for exclusion for the study were as follows: (1) pregnant women with irregular cycles with unknown date of LMP; (2) renal pelvic dilatation of 5 mm or greater at any GA; (3) multiple pregnancies; (4) renal anomalies; (5) anomalous fetus; (6) a history of diabetes or hypertension in pregnancy; and (7) failure to give consent. 

Method of selection

All patients who presented to the antenatal clinic at the Credence Hospital, Thiruvananthapuram, Kerala, India during the study period and satisfied the inclusion criteria were included in the study until the sample size target was attained.

Intervention

Only routine antenatal ultrasound was conducted. No other interventions were undertaken specifically for the study.

Data collection

The ultrasound was performed with an ultrasound machine (Voluson E10, GE Healthcare, Chicago, IL) using a 5 MHz curvilinear transducer, with the patient in the supine position. Good acoustic coupling was obtained using synthetic ultrasound gel. Only a single point of measurement was taken for all the participants. Right kidney length (RKL) and left kidney length (LKL), BPD, HC, AC, and FL were measured.

The planes used for measuring BPD and HC were sectioned through the third ventricle and thalamus. The cavum septi pellucidum should be visible in the anterior portion of the brain and the tentorial hiatus in the posterior portion of the brain. For BPD, the cursors were positioned from the outer edge of the near calvarial wall to the inner edge of the far calvarial wall. AC was taken from the outer-to-outer margin in the plane showing the umbilical vein perpendicular to the fetal spine and the stomach bubble. FL was obtained by aligning the transducer to the long axis of the diaphysis. Measurement cursors were placed at the junction of the cartilaginous epiphysis and bone. Fetal kidneys are located on either side of the spine and the adrenal gland is located close to the upper pole of the kidneys anatomically. Kidneys can be identified by moving the probe caudally in the transverse section just below the level for abdominal circumference measurement. Once the kidneys were located, the probe was rotated longitudinally until the full length of the kidney was calculated.

The adrenals were identified and were excluded. FKL measurements for both kidneys were made after identifying both the upper pole and the lower pole; the longest pole-to-pole measurement was then taken. The mean values of RKL and LKL were recorded as the FKL. The primary outcome was the FKL measurement from 24 weeks of gestation and the correlation of RKL, LKL, and FKL with GA.

Statistical analysis

The data were generated using a Microsoft Excel sheet. The RKL, LKL, and FKL were separately correlated with actual GA derived from the first-trimester crown-rump length detected by ultrasound. The linear relationship between continuous variables was computed using the Pearson correlation coefficient (r) and its significance was tested using a linear regression t-test. Simple and multiple linear regression analyses were performed to estimate beta coefficients (slope). The effectiveness of a model was determined by coefficient of determination (R^2^). Statistical analysis was performed using IBM SPSS Statistics version 22.00 (IBM Corp., Armonk, NY). A p-value <0.05 was considered to be statistically significant.

## Results

We enrolled 150 antenatal women above 24 weeks of gestation who presented to our antenatal clinic from August 1, 2022, to January 31, 2023 and met the inclusion criteria. The mean age of the study population was 29.23 ±4.53 years (range: 19-41 years). The mean GA of subjects was 33.18 ±3.18 weeks (Table 1). The short GA was 24 weeks and the longest was 39 weeks. Table 2 presents the descriptive data for RKL, LKL, and mean FKL at various gestational ages. The mean RKL was 33.03 ±3.09 mm with a minimum of 25 mm at 24 weeks and a maximum of 39 mm at 39 weeks. The mean LKL was 34.04 ±3.18 mm with a minimum of 26 mm at 24 weeks of gestation and a maximum of 40 mm at 39 weeks. We found no significant difference between right and left kidneys. The mean FKL was 33.51 ±3.15 mm at a mean GA of 33.41 ±3.18 weeks.

**Table 1:** Demographic data SD: standard deviation

Variable	Mean	SD	Minimum	Maximum
Age, years	29.23	4.53	19	41

**Table 2:** RKL, LKL, and FKL values at various gestational ages FKL: fetal kidney length; LKL: left kidney length; RKL: right kidney length; SD: standard deviation

Gestational age, weeks	N	LKL, mm		RKL, mm			FKL, mm	
		Mean	SD	Mean		SD	Mean	SD
24	1	25.5	0.0	24.5	0.0		25.0	0.0
26	1	26.5	0.0	25.5	0.0		26.0	0.0
27	2	27.6	0.84	26.9	1.27		27.25	1.06
28	6	28.68	0.94	27.62	0.86		28.15	0.56
29	5	29.84	0.68	29.14	0.48		29.48	0.21
30	11	31.00	0.93	29.78	0.92		30.36	0.68
31	17	31.66	1.21	30.58	0.69		31.10	0.71
32	25	32.66	0.69	31.59	1.02		32.09	0.68
33	11	33.76	0.85	32.89	1.21		33.19	0.67
34	15	34.34	0.68	33.56	0.76		33.95	0.57
35	6	35.73	0.94	34.72	0.65		35.22	0.55
36	16	36.61	0.87	35.38	0.70		35.98	0.64
37	18	37.53	0.65	36.60	0.81		37.06	0.60
38	12	38.59	0.44	37.59	0.82		38.09	0.53
39	4	39.28	0.26	38.18	0.78		38.72	0.48
Total	150							

**Figure 1:**
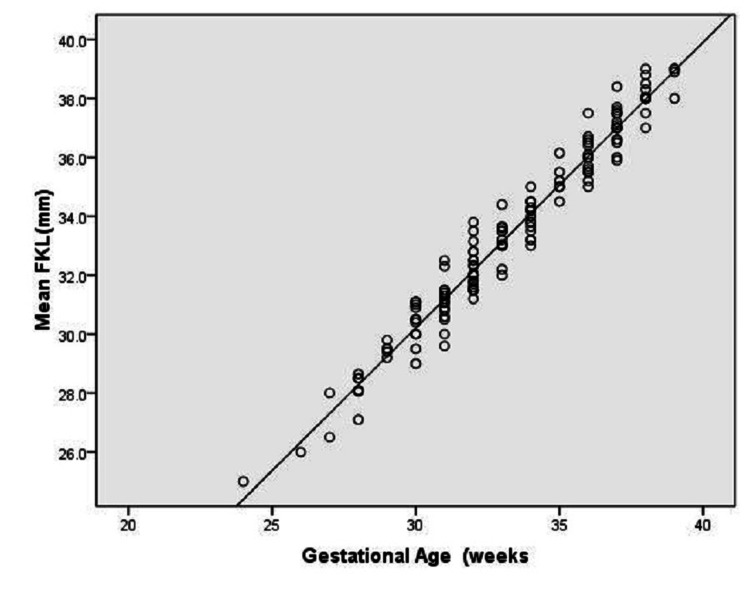
Scatter plot showing the correlation between mean FKL and GA FKL: fetal kidney length; GA: gestational age

We observed a strong correlation between mean FKL and GA (r=0.981) (Figure 1). The coefficient of determination was 96.2%, which reflects the proportion of variation in the dependent variable explained by the independent variable. The regression equation was as follows: GA = 1.13+0.97*FKL.

Linear regression analysis of the independent variables in predicting the gestational age has shown that fetal kidney length has the highest accuracy, with a standard error (SE) of 9.83 days, followed by FL, with an SE of 10.71 days. The most inaccurate was HC with an SE of 14.05 days (Table 3) The SE of the estimate measures the accuracy of predictions. On comparing the accuracy of various models combining various indices, the most accurate model was that combining FKL with BPD, HC, AC, and FL, with an SE of 8.09 days (Table 4).

**Table 3:** Linear regression equations defining the relationship between GA and various indices *P-value was tested using a linear regression t-test AC: abdominal circumference; AIC: Akaike information criterion; BPD: biparietal diameter; FDL: femur diaphysis length; GA: gestational age; HC: head circumference; KL: kidney length; SE: standard error

Parameter	Intercept		Slope		P-value*	R^2^	AIC	SE_pred_
	Estimate	SE	Estimate	SE				
KL, mm	0.148	0.546	0.992	0.016	<0.001	96.2	1687.85	9.83
BPD, mm	1.316	1.353	0.422	0.016	<0.001	81.6	1719.12	13.6
HC, mm	-3.662	1.495	0.125	0.005	<0.001	80.7	1385.25	14.05
AC, mm	4.857	0.949	0.100	0.003	<0.001	86.0	1569.47	11.93
FDL, mm	2.889	0.895	0.481	0.014	<0.001	88.8	1458.47	10.71

**Table 4:** The relationship between indices included and the precision with which GA is estimated between 24 and 39 weeks of gestation AC: abdominal circumference; AIC: Akaike information criterion; BPD: biparietal diameter; FL: fetal length; GA: gestational age; HC: head circumference; KL: kidney length; SE: standard error

Indices included	Regression equation	R^2^	AIC	SE_pred_,days
FL, KL	GA=0.069+0.086FL+0.833KL	92.5	1346.16	8.97
FL, KL, BPD	GA=0.408+0.074FL+0.797KL+0.029BPD	93.6	1287.68	8.22
FL, KL, HC	GA=0.596+0.071FL+0.804KL+0.009HC	93.6	1291.52	8.14
FL, KL, AC	GA=0.151+0.059FL+0.766KL+0.014AC	92.7	1352.89	9.18
FL, KL, BPD, HC	GA=0.601+0.070FL+0.795KL+0.017BPD +0.005HC	93.6	1268.71	8.74
FL, KL, BPD, AC	GA=0.30+0.057FL+0.761KL+0.007BPD +0.013AC	93.1	1314.45	8.56
FL, KL, BPD, HC, AC	GA=0.051+0.046FL+0.761KL+0.005BPD +0.011AC+0.002HC	93.1	1311.28	8.09

## Discussion

The accurate estimation of GA has a vital role in obstetric care. It is crucial for planning procedures like amniocentesis and the termination of high-risk pregnancies. The ideal scenario is a first-trimester estimation of GA by crown-rump length. However, in low-resource settings when women book later in pregnancy, other measures have to be conducted. BPD and FL predict with a margin of error of 6-10 days between 12 and 24 weeks. This study aimed at measuring kidney length after 24 weeks of gestation to assess GA. We were able to appropriately measure kidney length in all positions and at all GAs. Cohen et al. [6] have noted some difficulty when tests were done in the prone position. Similar observations were made by Duval et al. [13,14] for patients in the breech and vertex presentations with the back facing laterally or posteriorly. Easy identification of kidneys was reported by Konje et al. [7] by manipulation of transducer position and angle of insonation relative to the kidney plane.

In the present study, FKL at a particular GA corresponded well: 26 mm at 26 weeks, 28 mm at 28 weeks, 35 mm at 35 weeks, and 39 mm at 39 weeks. Bertagneli et al. [15] and Lawson et al. [16,17] found similar results, suggesting a rule of thumb that renal length in mm corresponds to GA in weeks. This means that anyone can predict the GA if the mean fetal length is known, without the aid of any specialized software. A previous study showed a significant correlation between the period of gestation and mean FKL [12] (r=0.94, p<0.05). In our study, the outcome was similar, with a correlation coefficient of 0.981. Other parameters measured in our study also showed a positive correlation between FKL and period of gestation (r=0.90 for BPD, r=0.898 for HC, r=0.928 for AC, and r=0.942 for FL). Our study shows that FKL can be used as an independent or additional parameter to determine GA.

Linear regression analysis of individual variables showed that FKL is the most accurate parameter to predict GA (SE: -9.83 days). For other indices, the SE was as follows: 13.6 days for BPD, 14.05 days for HC, 11.93 days for AC, and 10.71 days for FL. Konje et al. [10] obtained an SE of 10.29 days for FKL, 11.62 days for BPD, 11.19 days for HC, 14.54 days for AC, and 10.96 days for FL. Kansaria et al. [18] reported an SE of 9.17 days for FKL, 10.99 days for BPD, 11.14 days for AC, and 10.29 days for FL. In both these studies, FKL had the highest accuracy in predicting the GA followed by FL, similar to our study. The most inaccurate parameter in predicting GA in our study was HC, whereas it was AC in other studies, although this difference could not be explained.

The stepwise regression analysis between BPD, HC, AC, FL, kidney length, and period of gestation shows that a model including all the above five variables had the highest precision in estimating the period of gestation, with an accuracy of 8.09 days. The study by Konje et al. reported a similar outcome, with an accuracy of 8.48 days when all the five parameters were combined [10]. Sharma et al. highlighted that the measurement of GA and the timing of pregnancy are crucial components of the numerous risk models used to predict the likelihood of preeclampsia in FGR and preterm births [19].

Limitations of the study

As the current study adopted a cross-sectional design, we could not assess the rate of increase in FKL with the progression of GA. Also, we could not ensure that a sufficient sample size was recruited for each GA starting from 24 weeks.

Strengths of the study

All follow-up data were verified to be accurate and it was ensured that only those samples with complete information were used in the data analysis. The same individual performed all sonographic measures, and the same observer analyzed the research outcomes.

## Conclusions

Based on our findings, the pregnancies of people who booked late in pregnancy or those with uncertain dates could be dated accurately by measuring FKL. As it is the most accurate individual parameter for the estimation of the period of gestation in the late second and third trimesters, it can be incorporated into various models for the estimation of the period of gestation. However, our study found that the best model for predicting GA was the one that included all the five following parameters: BPD, HC, AC, FL, and kidney length.

## Figures and Tables

**Figure 2:**
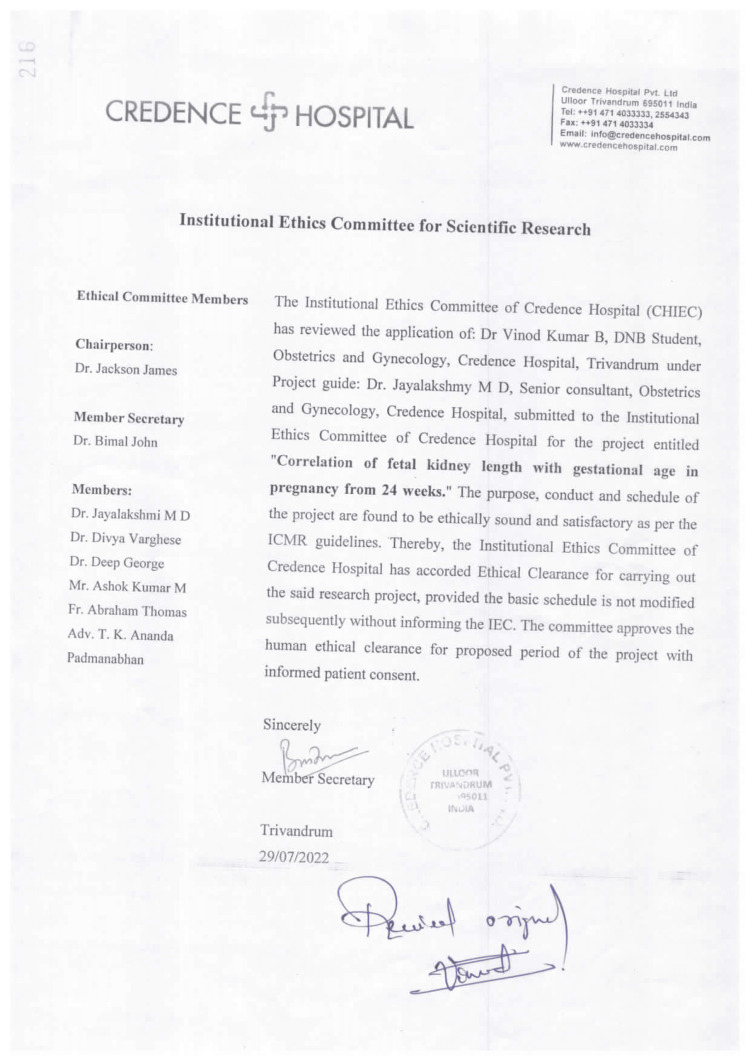
Ethical Committee approval letter - page 1

**Figure 3:**
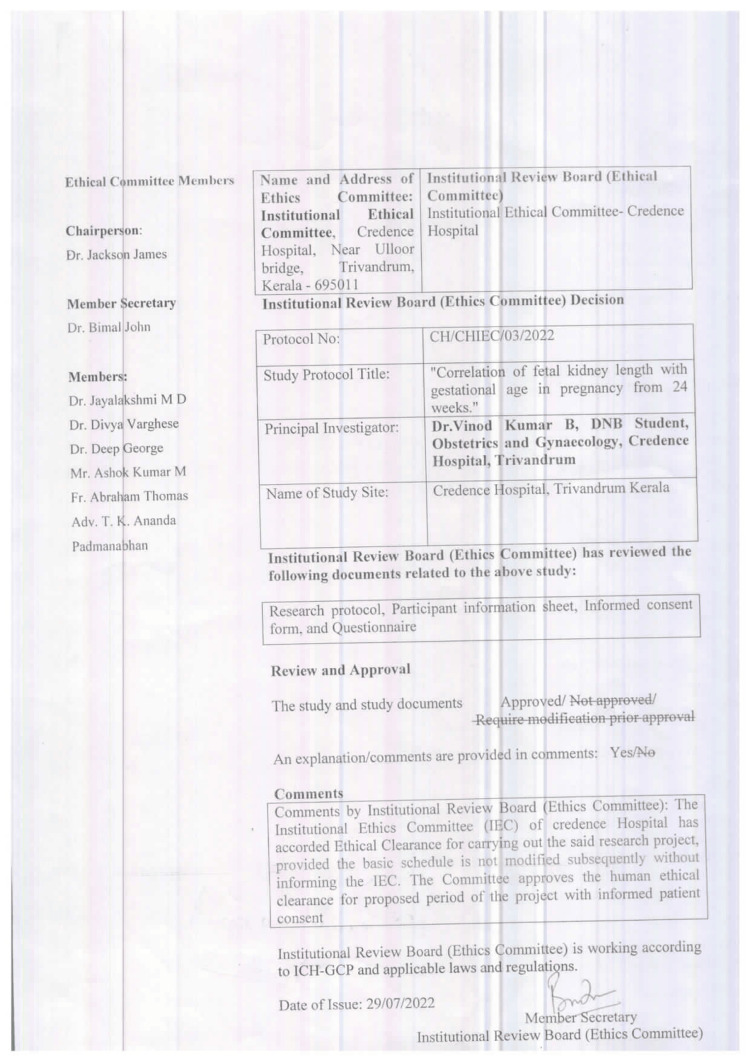
Ethical Committee approval letter - page 2
